# Identifying populations with chronic pain in primary care: developing an algorithm and logic rules applied to coded primary care diagnostic and medication data

**DOI:** 10.1186/s12875-023-02134-1

**Published:** 2023-09-11

**Authors:** Nasrin Hafezparast, Ellie Bragan Turner, Rupert Dunbar-Rees, Amoolya Vusirikala, Alice Vodden, Victoria de La Morinière, Katy Yeo, Hiten Dodhia, Stevo Durbaba, Siddesh Shetty, Mark Ashworth

**Affiliations:** 1 Outcomes Based Healthcare, 11-13 Cavendish Square, Marylebone, London, W1G 0AN UK; 2 Public Health Directorate, London Borough of Lambeth, Lambeth Civic Centre, 5th Floor, 2 Brixton Hill, London, SW2 1RW UK; 3 School of Life Course and Population Sciences, King’s College London, Guy’s Campus, Addison House, London, SE1 1UL UK

**Keywords:** Primary care, Long term conditions, Chronic pain, Electronic health record data

## Abstract

**Background:**

Estimates of chronic pain prevalence using coded primary care data are likely to be substantially lower than estimates derived from community surveys. Most primary care studies have estimated chronic pain prevalence using data searches confined to analgesic medication prescriptions. Increasingly, following recent NICE guideline recommendations, patients and doctors opt for non-drug treatment of chronic pain thus excluding these patients from prevalence estimates based on medication codes. We aimed to develop and test an algorithm combining medication codes with selected diagnostic codes to estimate chronic pain prevalence using coded primary care data.

**Methods:**

Following a scoping review 4 criteria were developed to identify cohorts of people with chronic pain. These were (1) people with one of 12 (‘tier 1’) conditions that almost always results in the individual having chronic pain (2) people with one of 20 (‘tier 2’) conditions included when there are also 3 or more prescription-only analgesics issued in the last 12 months (3) chronic neuropathic pain, or (4) 4 or more prescription-only analgesics issued in the last 12 months. These were translated into 8 logic rules which included 1,932 SNOMED CT codes.

**Results:**

The algorithm was run on primary care data from 41 GP Practices in Lambeth. The total population consisted of 386,238 GP registered adults ≥ 18 years as of the 31st March 2021. 64,135 (16.6%) were identified as people with chronic pain. This definition demonstrated notably high rates in Black ethnicity females, and higher rates in the most deprived, and older population.

**Conclusions:**

Estimates of chronic pain prevalence using structured healthcare data have previously shown lower prevalence estimates for chronic pain than reported in community surveys. This has limited the ability of researchers and clinicians to fully understand and address the complex multifactorial nature of chronic pain. Our study demonstrates that it may be possible to establish more representative prevalence estimates using structured data than previously possible. Use of logic rules offers the potential to move systematic identification and population-based management of chronic pain into mainstream clinical practice at scale and support improved management of symptom burden for people experiencing chronic pain.

**Supplementary Information:**

The online version contains supplementary material available at 10.1186/s12875-023-02134-1.

## Background

 Chronic pain is usually defined as pain lasting for more than 3 months [1]. There are many causes. Sometimes chronic pain is a feature of an underlying Long Term Condition (LTC) such as osteoarthritis, endometriosis or cancer, in which case the pain is described as chronic secondary pain. In other situations, chronic pain has no clear underlying cause and is termed chronic primary pain; examples include fibromyalgia, chronic daily headache, complex regional pain syndrome, irritable bowel syndrome. Chronic primary pain is likely to have a basis in a mix of physiological, psychological and social factors. Approximately one third of people with chronic pain have a musculo-skeletal (MSK) cause, a third report no underlying LTC, 15% have a mental health disorder with smaller proportions reporting less common underlying causes [2]. Mental health problems may be linked both to causes and consequences [3].

Prevalence estimates for chronic pain vary widely. The Health Survey for England (2017), a community survey, found that 34% of adults had chronic pain, a prevalence that increased to 53% of adults aged 75 years and over [2]. In one of the few systematic reviews of chronic pain prevalence, 19 studies were identified with data from almost 140,000 people producing a pooled prevalence estimate of 43.5% [4].

Estimates based on primary care data searches are likely to produce lower prevalence values than community surveys, resulting in considerable underestimation of true prevalence, especially if coded data searches are confined to analgesic medication prescriptions such as non-steroidal anti-inflammatory analgesics (NSAIDs) or opioids, or diagnostic codes for simply ‘chronic pain’. In a UK study based on 400,000 adult patients using Clinical Practice Research Datalink (CPRD) data, the registered adult population (≥ 18 years) prevalence of chronic pain was 10.1% [5]. Identification of chronic pain patients in this study relied on computerised searches for analgesic medication developed by the Primary Care Unit, University of Cambridge [6]: 4 or more Prescription Only Medications (POMs) in the last 12 months with exclusions for analgesics with dual indication such as epilepsy (in patients with an ‘epilepsy’ coding). Similarly, a study based on Scottish primary care data from 1.75 million patients of all ages, again confined to medication data, noted a prevalence of 7.2% for ‘painful condition’ [7].

Although prevalence shortfalls are to be expected for many LTCs using coded primary care data searches when compared to community survey data, the ‘prevalence gap’ for chronic pain is likely to be large. This is because of a lack of consistent ‘chronic pain’ coding in primary care records [8], the fact that chronic pain is often the result of a diverse range of often unrelated underlying conditions and that many patients and doctors increasingly opt for non-drug management of their pain, as currently recommended by NICE guidelines [1]. We aimed to develop and conduct initial testing of an algorithm to detect patients at risk of chronic pain using coded primary care data, combining medication codes with selected diagnostic codes including codes for ‘chronic pain’, and for conditions known to be associated with chronic pain.

## Methods

Chronic pain was included as one of 32 Long Term Conditions (LTCs) as part of Guy’s and St Thomas’ Charity’s Multiple Long Term Conditions (MLTC) programme [9, 10]. Each LTC was identified using a set of clinical and medication codes (Read, SNOMED CT and EMIS codes). To identify patients with chronic pain, a set of clinical codes and logic rules (business rules) were developed and applied to anonymised person-level primary care data.

Initially, a scoping review was undertaken to understand how chronic pain is defined clinically by 16 different national and international organisations (Table S1, Supplementary File). This included the Faculty of Pain Medicine of the Royal College of Anaesthetists UK, the National Institute for Health and Care Excellence (NICE) and the American Chronic Pain Association. We reached a consensus definition of chronic pain, based on the literature: ‘a persistent or recurrent pain, lasting 3 months or longer’.

A further scoping review was undertaken to identify sources which have used conditions, codes or logic rules to identify people with chronic pain from electronic health record data. This led to the development of four criteria compiled into a unified definition to include any individual who met one or more of these four criteria.

In a systematic review by Carreira et al., definitions and combinations of codes were used to identify chronic pain (amongst other conditions) using electronic health records from primary care databases in the UK [11]. Eight studies were found which defined chronic pain; and of these, four included Read code lists for chronic pain [12–15]. Based on the lists of Read codes across these published sources (approximately 3000 Read codes), a list of ‘tier 1’ and ‘tier 2’ conditions was derived, discussed and agreed by the clinicians among the co-authors (RD-R, HD, MA). Tier 1 conditions were those that would almost always result in chronic pain. Tier 2 conditions were those (typically on the more severe end of their spectrum of severity) that could result in chronic pain. Coded painful conditions which might be either acute or chronic were only included in Tier 2 if there was additional evidence of longer term analgesic prescriptions which would indicate clinically significant chronic pain. In addition, Read codes for dysmenorrhoea, migraine and endometriosis conditions, which were not in the original list of Read codes, were included in tier 2 [16].

### Criteria 1 -- conditions very strongly associated with chronic pain

Includes all people with a condition or diagnosis that almost always results in the individual having chronic pain. These 12 conditions have been labelled as ‘tier 1’ conditions.

### Tier 1 conditions

- Ankylosing spondylitis
- Chronic low back pain
- Chronic osteomyelitis
- Chronic pain
- Complex Regional Pain syndrome
- Fibromyalgia
- Fibrositis
- Periostitis
- Rheumatic pain
- Rheumatism
- Rheumatoid arthritis
- Still’s disease

### Criteria 2 -- conditions associated with chronic pain

Includes all people with a condition that could result in chronic pain, and therefore only included when there are also 3 or more prescriptions issued for a prescription-only analgesia (as per those used in Criteria 4, below) in the last 12 months. These 20 broad condition groups have been labelled as ‘tier 2’ conditions.

### Tier 2 conditions

- Arthrosis/ arthritis/ arthralgia/ arthropathy (excl. reactive/ transient)
- Cervicocranial/ cervicobrachial syndrome
- Coccygodynia
- Connective tissue disorders
- Dysmenorrhoea
- Endometriosis
- Familial chondrocalcinosis
- Migraine
- Myositis (excl. infective causes)
- Osteoarthritis
- Osteochondritis
- Osteoporosis (incl. fragility fracture and collapsed vertebra)
- Pain/ache of different body parts/general aches & pains
- Pathological fracture
- Polymyalgia/PMR/GCA
- Radiculopathy (incl. cauda equina compression)/ radiculitis
- Sciatica
- Spinal stenosis
- Spondylopathy/ spondylosis/ spondylolisthesis
- Systemic Lupus Erythematosus

### Criteria 3 -- chronic neuropathic pain

**Fig. 1:**
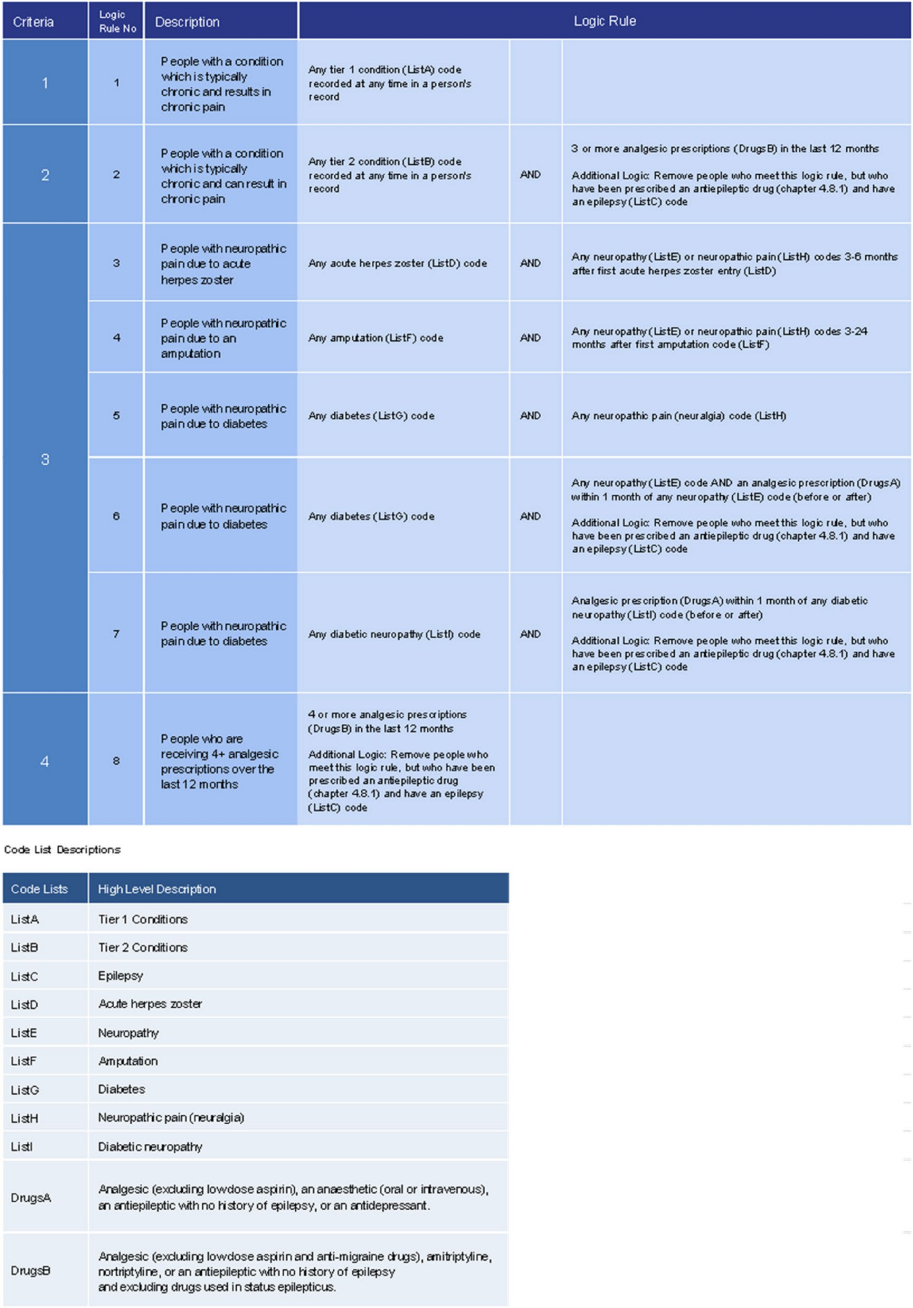
Overarching logic for population: all people aged 18 and over who are currently registered with a general practitioner

Three publications were identified with definitions for neuropathic pain as a form of chronic pain [17–19]. These were collated for this criterion which includes people with neuropathic pain as a result of the following 4 conditions. This results in 5 logic rules (see Fig. 1).

- Trigeminal neuralgia;
- Post herpetic neuralgia, including people with neuropathy or neuropathic pain 3-6 months following an episode of acute herpes zoster;
- Phantom limb pain, including people with neuropathy or neuropathy pain 3-24 months following an amputation;
- Painful diabetic neuropathy, including people with a diabetic neuropathy and a neuropathic pain treatment*, or diabetes and neuropathic pain (coded separately).

*A neuropathic pain treatment was defined as an analgesic (excluding low dose aspirin), an anaesthetic (oral or intravenous), an antiepileptic in patients with no history of epilepsy, or an antidepressant.

### Criteria 4 -- regular analgesic prescriptions

Includes people who have had 4 or more prescriptions issued for a prescription-only analgesia in the last 12 months. The original source of this definition is the Cambridge Primary Care Unit [6] and is the same list of analgesics used for tier 2 conditions. It includes the following 4 categories of medication:

- Non-opioid analgesics (except low dose aspirin);
- Opioid analgesics; neuropathic pain analgesics;
- Tricyclic antidepressants (amitriptyline and nortriptyline only);
- Antiepileptic drugs (except if the person has a diagnosis of epilepsy).

To enable the definition made up of these 4 criteria to be translated into queries or searches and applied to primary care data, they needed to be further divided into individual logic rules. The logic includes combinations of clinical diagnostic codes and prescription codes, as well as timeframes in which specific prescriptions are issued and conditions are diagnosed. Each component of the logic rules refers to a list of SNOMED CT clinical codes (Lists A-I) or BNF drug chapters (Drugs A-B) (Fig. 1).

Further expansion and refinement of the SNOMED CT codes: following recent changes in clinical coding classifications used in GP IT systems, from Read to SNOMED CT, a total of 780 Read codes used in this definition were translated into 552 SNOMED CT codes through Read to SNOMED CT mapping tables, followed by a clinical review of all SNOMED CT codes found through this method. In addition, SNOMED CT searches [20] were carried out for the conditions listed in the criteria resulting in the addition of a further 1,380 SNOMED CT codes. These searches produced a final list of 1,932 SNOMED CT codes used together with the eight logic rules (Fig. 1).

## Results

### Data source

We aimed to validate the ‘chronic pain’ SNOMED CT codes and algorithms on a primary care dataset. We used primary care data from Lambeth DataNet, an anonymised database containing data from all general practices in Lambeth (n = 41), an inner-city, multi-ethnic and relatively young population [21–23]. The data extracted covered the period 1st April 2005 to 31st March 2021, and included all registered adults ≥ 18 years with one or more of the 32 LTCs as of 31st March 2021 [8]. Approximately 4.6% of patients had ‘Informed Dissent’ codes in their Electronic Health Record (EHR) and their data could not be included in this analysis. The total sample population consisted of 386,238 currently GP registered adults ≥ 18 years as of the 31st March 2021. Since this was part of a wider project on multimorbidity and health inequalities, the prevalence of chronic pain was compared with other LTCs and stratified by demographic characteristics. Ethnicity was characterised using the five national Census codes [24]. Social deprivation was based on the Index of Multiple Deprivation 2019, stratified into national quintiles [25].

**Fig. 2:**
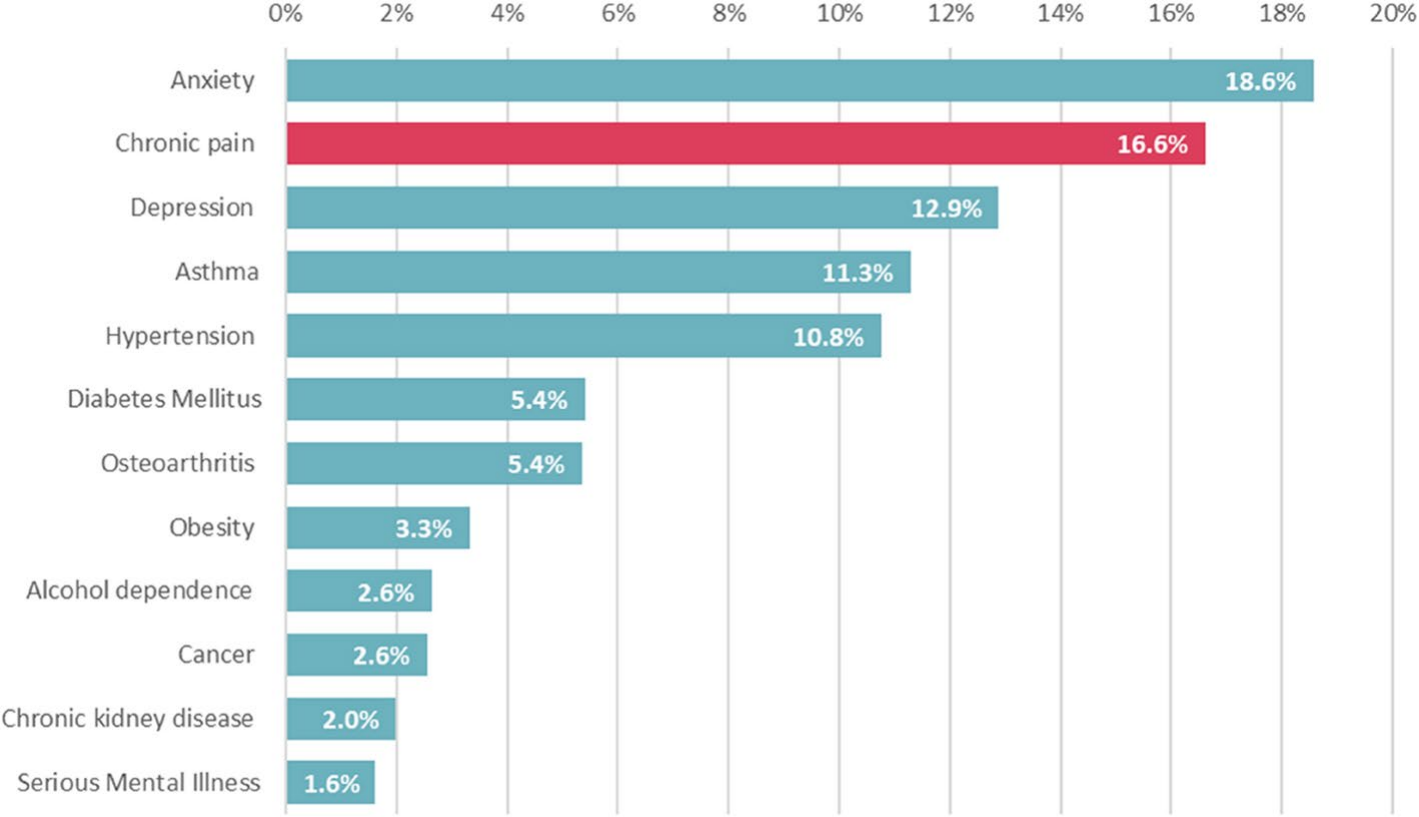
Prevalence of top 12 LTCs in all adults registered at a GP practice in Lambeth

An initial pilot version of the algorithm (developed prior to the SNOMED CT codes which underwent further expansion and refinement) was developed and run on the longitudinal data source. The chronic pain logic rules and codes were applied to the whole period of the dataset and a snapshot taken as of 31st March 2021. A total of 183,092 (47.4%) had a record of one or more of the 32 LTCs, and a total of 64,135 (16.6%) were identified with chronic pain (Fig. 2.

**Fig. 3:**
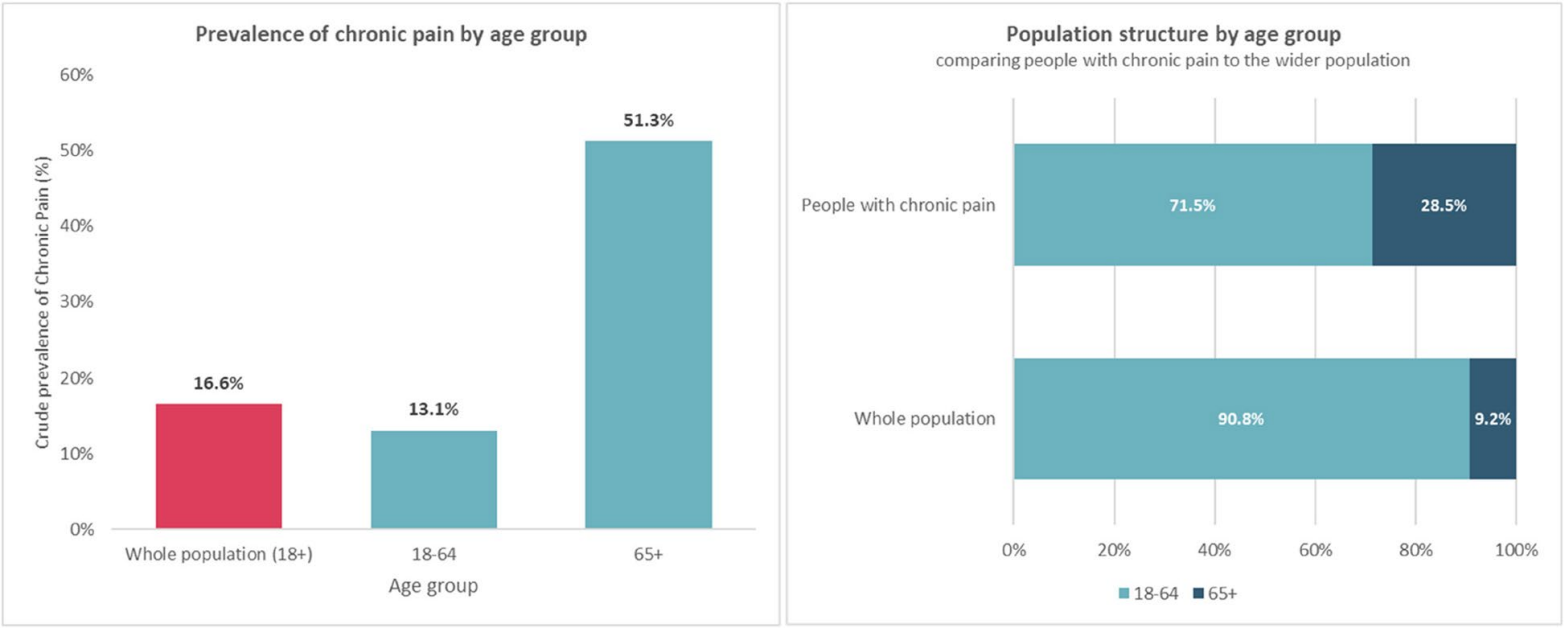
Prevalence of chronic pain by age group and population structure by age group comparing people with chronic pain to the wider population

**Fig. 4:**
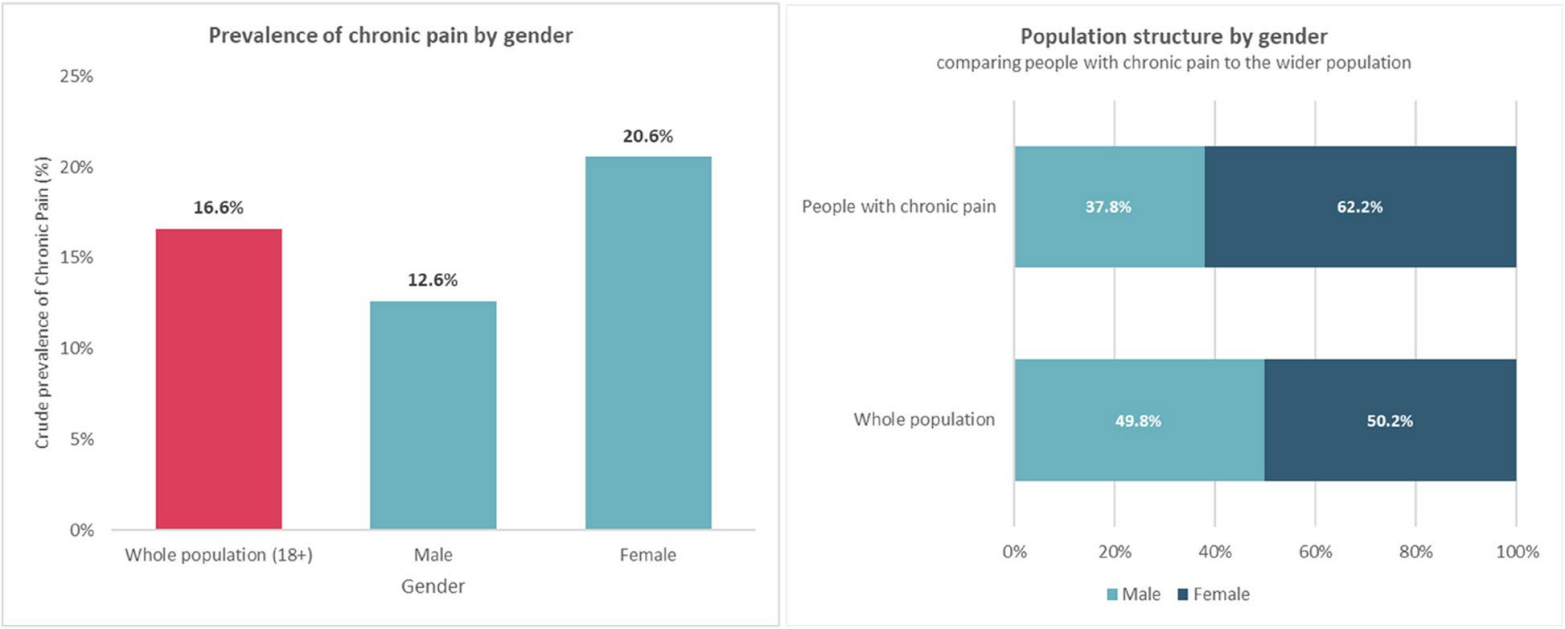
Prevalence of chronic pain by gender and population structure by gender comparing people with chronic pain to the wider population

**Fig. 5:**
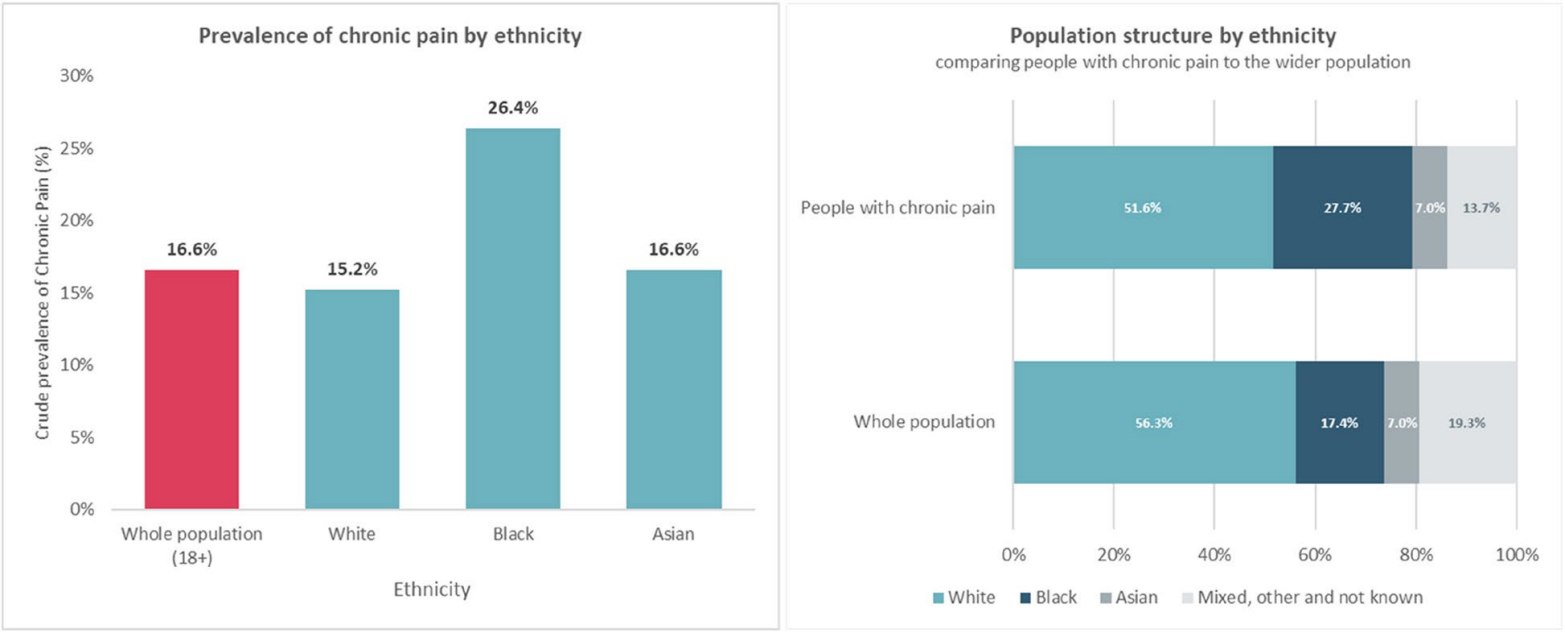
Prevalence of chronic pain by ethnicity and population structure by ethnicity comparing people with chronic pain to the wider population

**Fig. 6:**
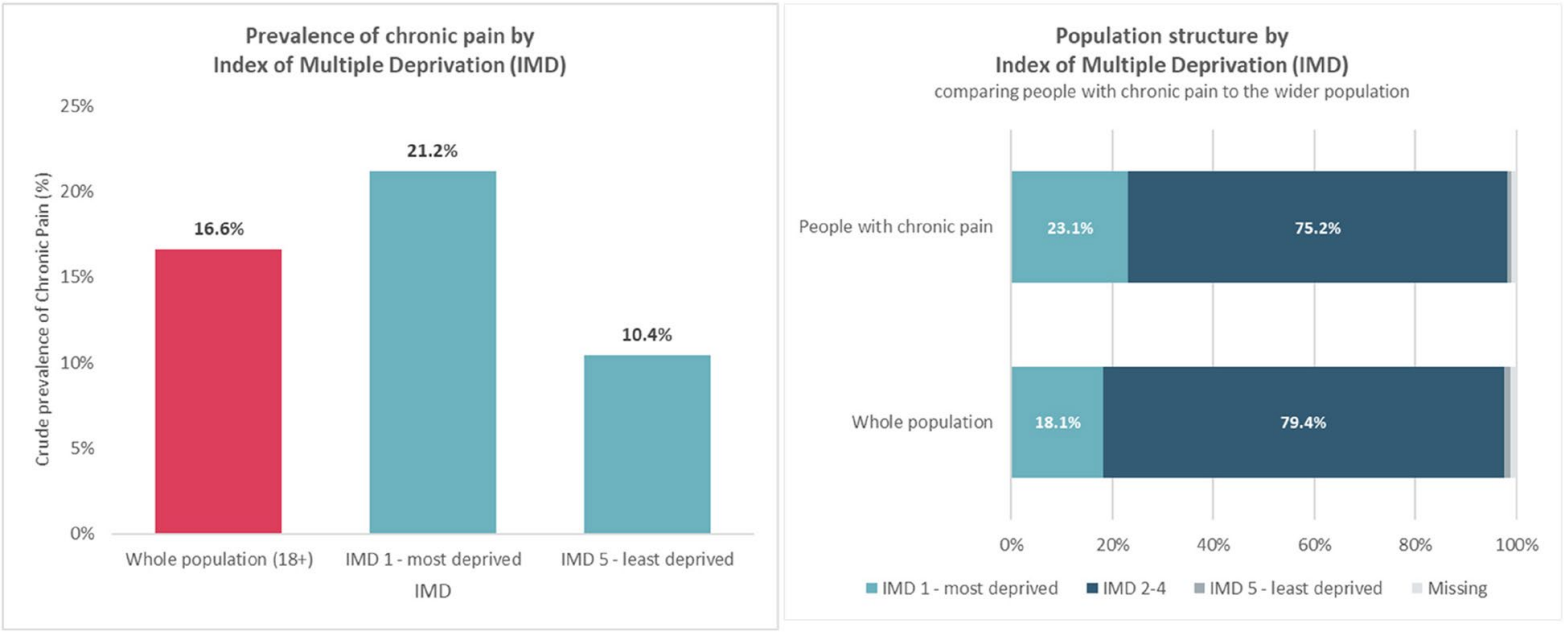
Prevalence of chronic pain by most deprived and least deprived (IMD) and population structure by deprivation comparing people with chronic pain to the wider population

The following results provide further socio-demographic data of the population identified by the chronic pain algorithm in relation to age (Fig. 3), gender (Fig. 4), ethnicity (Fig. 5), and deprivation (Fig. 6). Prevalence of chronic pain was higher in people aged 65 years and above (compared to people aged 18 to 64): 51.3% vs. 13.1%; females (compared to males): 20.6% vs. 12.6%; people of Black ethnicity (compared to people of White or Asian ethnicity): 26.4% vs. 15.2%; people in the most deprived IMD quintile nationally (compared to the least deprived quintile): 21.2% vs. 10.4%.

## Discussion

The consensus driven algorithm for chronic pain was based on four overarching criteria consisting of a total of 1,932 SNOMED CT codes used across 8 logic rules. These codes and logic rules were applied to primary care data extracted from the Electronic Health Record. Based on this method and results, the overall prevalence of chronic pain in adults currently registered in primary care was 16.6%. The method was feasible; it had face validity as observed prevalence was higher than in other studies based on medication prescribing alone; and followed a similar pattern to reported chronic pain prevalence according to gender, ethnicity and deprivation [2]. This definition has contributed to the broader study of health inequalities demonstrating notably high rates in Black ethnicity females, and higher rates in the most deprived, and older population. Although our findings are likely to be under-estimates of true community prevalence of chronic pain, this detailed methodology has produced higher primary care prevalence estimates than those based on analgesic prescribing alone [5, 7]. As such, these findings highlight the importance of chronic pain which is associated with high rates of primary care resource utilisation [26]. Chronic pain is also likely to lead to substantial functional impairment and disability, although this is under-reported and difficult to quantify using primary care records [27].

### Strengths and limitations

It is important to note that the purpose of this work was to support data analysis that was considered ‘secondary use’; no patient identification was required or carried out to contact patients, for interventions or care. In addition, at a population level it was used to identify all people who had experienced chronic pain previously based on the above definition, not only those who are experiencing chronic pain currently. Therefore, this definition will only identify people who are at risk of chronic pain, and not only those who are currently experiencing chronic pain. Although this analysis revealed a significantly greater number of people at risk of chronic pain than previous approaches, there has as yet been no independent validation of this definition to establish how well the combined definition is successfully identifying those experiencing chronic pain currently.

It would be possible to refine the algorithm further to remove people who may have a tier 2 condition but have not met the prescribed analgesia thresholds in the most recent 12 month period, and the same for prescribed analgesia thresholds during the most recent 12 month period (criteria 4). This would have the effect of reducing the reported prevalence of people at risk of experiencing chronic pain but may increase the positive identification rate of those experiencing chronic pain currently. How such thresholds and logic are applied within the algorithm should largely be determined by each use case. These include how important it is to identify all people potentially experiencing chronic pain including a higher number of ‘false positives’, versus identifying a smaller number but with a greater likelihood of accurately identifying people currently experiencing chronic pain.

A further potential limitation relates to the fact that chronic pain has historically been considered a symptom of a range of underlying causes, rather than a ‘condition’ in its own right. One consequence of this is that the criteria above are not mutually exclusive, so a clear picture of which specific criteria above are most driving the chronic pain prevalence observed in this study and the interplay with other LTCs is complex. People may enter the chronic pain cohort through attribution to multiple criteria above. Understanding which of the above criteria are most important in identifying chronic pain would be highly complex and would ideally need to be undertaken as further research, once it is confirmed that people identified using coded primary care data are corresponding well to those identified by community surveys. On the other hand, inclusion of the broad diagnostic coding category of ‘chronic pain’ in our definition of a Tier 1 condition would be expected to capture a diversity of conditions through more granular SNOMED CT codes not specifically included elsewhere in Criteria 2–4 such as chronic pain related to a cancer diagnosis.

A further consequence of the chronic pain definition being made up of other LTC definitions is that the analysis of multiple long term conditions in the population becomes more complex. For example, all people with rheumatoid arthritis would be included in the multiple long term conditions cohort as they would have a flag for both ‘chronic pain’ and ‘rheumatoid arthritis’.

It is likely that our findings represent an underestimate of chronic pain prevalence for patients in primary care. Firstly, patients may fulfil our diagnostic criteria for chronic pain but not be identified as chronic pain patients because they opt for over-the-counter analgesic medication. Secondly, our exclusion criteria meant that some patients with both epilepsy and chronic pain who only met criteria 4 were excluded, since analgesics with anti-epileptic properties were excluded. Thirdly, patients prescribed SSRIs or duloxetine were not included because of uncertainty about whether primary indication was depression or chronic pain.

### Implications for practice

In order to test and implement this method of defining chronic pain, identifying people who fulfil these specific criteria for chronic pain and to contribute to clinical practice, this study has subsequently been translated into an EMISWeb Search (EMIS is the clinical software supplier for all general practices contributing data to Lambeth DataNet). This may support piloting, further validation and identification of those potentially at risk of chronic pain for clinical review by their GPs. Following confirmation of chronic pain by their GP, people could be offered interventions aligned to NICE guideline recommendations [1], acknowledging the importance of a holistic biopsychosocial approach to management the management of chronic pain and supporting approaches to self-management.

## Conclusions

Estimates of chronic pain prevalence in the community using structured healthcare data have previously shown lower prevalence estimates for chronic pain than community surveys. This has limited the ability of researchers and clinicians to fully understand and address the complex multifactorial nature of chronic pain in practice.

This study demonstrates that it may be possible to establish more representative prevalence estimates using structured data than has previously been possible. Using data of this kind, it may be more practical to continuously understand changes in prevalence of chronic pain, and explore potential drivers in specific groups of people more likely to experience chronic pain. This offers the potential to move systematic identification and population-based management of chronic pain into mainstream clinical practice at scale, and support improved symptom burden for people experiencing chronic pain. However, prevalence estimates of this kind will always depend on definitions and purpose. Future research should ideally focus on improving consistency of structured data definitions in chronic pain, and validating outputs of people flagged as at risk of experiencing chronic pain with patients directly.

### Supplementary Information

**Additional file 1.**

## Data Availability

The code lists and logic rules developed during the current study are available from the corresponding author on reasonable request.

## Declarations

All data for our study were extracted under the terms of both a legally binding Data Sharing Agreement (DSA) signed by each general practice; also, project-specific approval following submission of a Data Privacy Impact Assessment (DPIA), approved by both NHS Lambeth Clinical Commissioning Group and the Caldicott Guardian on 21st November 2019.

Caldicott Guardian approval required confirmation that all aspects of the study including ethical approval and patient consent exemptions were compliant with the following UK legislation: Data Protection Act 2018 (Chap. 2, Sects. 8 and 10, in clause 3.3); Health and Social Care Act 2012 (Sect. 261).

This study was exempt from ethical committee approval and the requirement to obtain individual patient consent. Exemptions for research studies using anonymised primary care data apply to studies fulfilling requirements specified by the national body, the NHS Health Research Authority (HRA) and summarised in the HRA Integrated Research Application System (IRAS) guidance to researchers: https://www.myresearchproject.org.uk/help/hlpcollatedqsg-iras.aspx. Specifically, written confirmation of exemption has been confirmed by the HRA on the following grounds: patients have been informed through the process termed ‘Fair Processing Notices’ (FPNs) that their health care data would be available for ‘secondary data analysis’ and given the option to opt-out of data sharing through the process termed ‘Informed Dissent’; all data provided to researchers was fully anonymised; all data extracted from general practices was Pseudonymised At Source (PAS); the pseudonymisation process was ‘one-way’ and could not be reverse engineered to obtain any personal identifiers; all Patient Identifiable Data (PID) was removed; ‘low number suppression’ was applied to all research findings, ensuring that data would not be displayed with ≤ 10 patients in any data category (personal communication from HRA, 29th September 2017).

## References

[1] National Institute for Health and Care Excellence (NICE) (2021) NG193: Chronic pain (primary and secondary) in over 16s: assessment of all chronic pain and management of chronic primary pain. https://www.nice.org.uk/guidance/ng193. Accessed 08 Dec 2022. 33939353

[2] Chronic Pain in Adults. 2017. Health Survey for England. London: Public Health England; 2017. https://assets.publishing.service.gov.uk/government/uploads/system/uploads/attachment_data/file/940858/Chronic_Pain_Report.pdf. Accessed 08 Dec 2022.

[3] Bondesson E, Larrosa Pardo F, Stigmar K, Ringqvist Ã, Petersson IF, Jöud A (2018). Comorbidity between pain and mental illness – evidence of a bidirectional relationship. Eur J Pain.

[4] Fayaz A, Croft P, Langford RM, Donaldson LJ, Jones GT (2016). Prevalence of chronic pain in the UK: a systematic review and meta-analysis of population studies. BMJ Open.

[5] Cassell A, Edwards D, Harshfield A, Rhodes K, Brimicombe K, Payne R (2018). The epidemiology of multimorbidity in primary care: a retrospective cohort study. Br J Gen Pract.

[6] CPRD @ Cambridge – codes lists (GOLD). Primary Care Unit, University of Cambridge, version 1.1 lists. 2018. https://www.phpc.cam.ac.uk/pcu/research/research-groups/crmh/cprd_cam/codelists/v11/. Accessed on 08 Dec 2022.

[7] Barnett K, Mercer SW, Norbury M, Watt G, Wyke S, Guthrie B (2012). Epidemiology of multimorbidity and implications for health care, research, and medical education: a cross-sectional study. Lancet.

[8] Foell J, Carnes D, Homer K, Taylor S (2014). Developing and implementing electronic search strategies to recruit patients with chronic musculoskeletal pain in primary care databases. Prim Health Care Res Dev.

[9] Hafezparast N, Turner EB, Dunbar-Rees R (2021). Adapting the definition of multimorbidity – development of a locality-based consensus for selecting included Long Term Conditions. BMC Fam Pract.

[10] Urban Health. Multiple long term conditions. London: Impact on Urban Health. 2022. https://urbanhealth.org.uk/our-work/multiple-long-term-conditions. Accessed 08 Dec 2022.

[11] Carreira H, Williams R, Strongman H, Bhaskaran K (2019). Identification of mental health and quality of life outcomes in primary care databases in the UK: a systematic review. BMJ Open.

[12] Campbell P, Shraim M, Jordan KP, Dunn KM (2022). In sickness and in health: A cross-sectional analysis of concordance for musculoskeletal pain in 13,507 couples. Eur J Pain.

[13] Mansfield KE, Sim J, Croft P, Jordan KP (2022). Identifying patients with chronic widespread pain in primary care. Pain.

[14] Ruigómez A, Rodríguez LA, Wallander MA, Johansson S, Joneset R (2006). Chest pain in general practice: incidence, comorbidity and mortality. Fam Pract.

[15] Wallander MA, Johansson S, Ruigómez A, Rodríguez LA. Unspecified abdominal pain in primary care: the role of gastrointestinal morbidity. Int J Clin Pract 2007;61:1663-70. 10.1111/j.1742-1241.2007.01529.x. Accessed 08 Dec 2022. 10.1111/j.1742-1241.2007.01529.x 17681003

[16] Kuan V, Denaxas S, González-Izquierdo A, Direk K, Bhatti O, Husain S (2022). A chronological map of 308 physical and mental health conditions from 4 million individuals in the English National Health Service. Lancet Digit Health.

[17] Hall GC, Carroll D, Parry D (2022). Epidemiology and treatment of neuropathic pain: the UK primary care perspective. Pain.

[18] Hall GC, Carroll D, McQuay HJ (2008). Primary care incidence and treatment of four neuropathic pain conditions: a descriptive study, 2002–2005. BMC Fam Pract.

[19] Hall GC, Morant SV, Carroll D, Gabriel ZL, McQuay HJ (2013). An observational descriptive study of the epidemiology and treatment of neuropathic pain in a UK general population. BMC Fam Pract.

[20] NHS Digital SNOMED CT Browser, International SNOMED. NHS Digital, 017. https://termbrowser.nhs.uk/. Accessed 31 Jan 2023.

[21] Bisquera A, Gulliford M, Dodhia H, Ledwaba-Chapman L, Durbaba S, Soley-Bori M (2021). Identifying longitudinal clusters of multimorbidity in an urban setting: A population-based cross-sectional study. Lancet Reg Health Eur.

[22] Bisquera A, Turner E, Ledwaba-Chapman L, Dunbar-Rees R, Hafezparast N, Gulliford M (2021). Inequalities in developing multimorbidity over time: A population-based cohort study from an urban, multi-ethnic borough in the United Kingdom. Lancet Reg Health Eur.

[23] Ledwaba-Chapman L, Bisquera A, Gulliford M, Dodhia H, Durbaba S, Ashworth M (2021). Applying resolved and remission codes reduced prevalence of multimorbidity in an urban multi-ethnic population. J Clin Epidemiol.

[24] Office for National Statistics. Statistical Bulletin. Ethnic group, England and Wales: Census 2021. Office for National Statistics, London. 2022. https://www.ons.gov.uk/peoplepopulationandcommunity/culturalidentity/ethnicity/bulletins/ethnicgroupenglandandwales/census2021. Accessed 31 Jan 2023.

[25] The English indices of deprivation 2019 (IoD2019). London: Ministry of Housing Communities & Local Government; 2019. https://assets.publishing.service.gov.uk/government/uploads/system/uploads/attachment_data/file/835115/IoD2019_Statistical_Release.pdf. Accessed 31 Jan 2023.

[26] Soley-Bori M, Bisquera A, Ashworth M (2022). Identifying multimorbidity clusters with the highest primary care use: 15 years of evidence from a multi-ethnic metropolitan population. Br J Gen Pract.

[27] Nahin RL, Sayer B, Stussman BJ (2019). Eighteen-year trends in the prevalence of, and health care use for, noncancer pain in the United States: data from the medical expenditure panel survey. J Pain.

