# Supplementary material for: Identifying populations with chronic pain in primary care: developing an algorithm and logic rules applied to coded primary care diagnostic and medication data
Source: BMC Prim Care. 2023 Sep 11;24:184. doi: 10.1186/s12875-023-02134-1 (PMC10494405; doi:10.1186/s12875-023-02134-1)
Supplement: Supplementary file 1 — Additional file 1. [file 12875_2023_2134_MOESM1_ESM.docx]

#### Supplementary File, Table S1.

| **Source** | **Chronic Pain Definition** |
| --- | --- |
| Merskey H, Bogduk N. Classification of chronic pain. 2nd ed. (Revised) Seattle: IASP Press, 1994.^1^ | Pain which persists past the normal time of healing (Bonica 1953). In practice this may be less than one month, or more often, more than six months. With nonmalignant pain, three months is the most convenient point of decision between acute and chronic pain, but for research purposes six months will often be preferred. |
| British Pain Society^2^ | Pain is an emotion experienced in the brain, it is not like touch, taste, sight, smell or hearing. It is categorised into acute pain - less than twelve weeks duration and chronic pain - of more than twelve weeks. |
| Faculty of Pain Medicine of the Royal College of Anaesthetists UK^3^ | Pain is usually described as acute (short term) or chronic (long term - usually more than three months). Chronic pain sometimes begins with an acute injury but the pain does not resolve as expected; often it is not clear how a chronic pain has started. Common types of chronic pain include low back pain, pain related to arthritis and pain related to injury to a nerve or other part of the nervous system (neuropathic pain). |
| NICE Guideline  Chronic Pain (primary and secondary) in over 16s: assessment of all chronic pain and management of chronic primary pain^4^ | Chronic pain (sometimes known as long-term pain or persistent pain) is pain that lasts for more than 3 months. Pain can be secondary to (caused by) an underlying condition (for example, osteoarthritis, rheumatoid arthritis, ulcerative colitis, endometriosis). Chronic pain can also be primary. This guideline is consistent with the ICD-11 definition of chronic primary pain. |
| Healthcare Improvement Scotland SIGN Clinical Guideline 136^5^ | Chronic pain is defined as pain that has been present for more than 12 weeks. |
| NHS Inform (Scotland)^6^ | Chronic or persistent pain is pain that carries on for longer than 12 weeks despite medication or treatment. |
| NHS Cornwall and Isles of Scilly Integrated Care System - Pain Services^7^ | Chronic pain is pain persisting after the healing process has occurred or in the absence of tissue injury. It is sometimes defined as pain lasting for more than 3 months. |
| Wigan Borough CCG (Opioid Prescribing for Chronic Pain Resource Pack)^8^ | Chronic pain refers to a continuous pain that persists beyond the expected time of healing or for longer than 3 months excluding cancer related pain and pain experienced at the end of life care. |
| Oxford University Hospitals NHS Foundation Trust (Optimise Pain Rehabilitation Unit)^9^ | Chronic or persistent pain is any pain that has lasted for three months or longer. |
| Institute for Chronic Pain (Education & Public Policy Think Tank)^10^ | Chronic pain has two characteristics that are different than acute pain. First, chronic pain lasts longer than six months. Second, and most importantly, chronic pain is pain that occurs in addition to the pain of the original health condition. In fact, the original, underlying condition may or may not have healed. It doesn't really matter. Chronic pain is pain that has become independent of the underlying injury or illness that started it all. |
| Institute of Medicine (US) Committee on Advancing Pain Research, Care, and Education. Relieving Pain in America.^11^ | Chronic pain, lasts more than several months (variously defined as 3 to 6 months, but certainly longer than “normal healing”) |
| The Pain Toolkit^12^ | Persistent pain (chronic or long-term pain) is pain that continues for a period of 3 months or more and may not respond to usual medical treatment. |
| American Society of Anesthesiologists: Chronic Pain^13^ | Chronic pain is pain that won’t go away, lasting three months or longer. Examples include arthritis in your knees, back, or neck that hurts most days; frequent migraine headaches; surgical pain that isn’t treated properly and lingers; and pain from muscle injuries that don’t heal correctly. |
| National Institute of Neurological Disorders and Stroke^14^ | Chronic pain is a medical disease that can be made worse by environmental and psychological factors. Chronic pain persists over a long period and can be challenging to manage. People with chronic pain often suffer from more than one painful condition. |
| Practical Pain Management (Journal)^15^ | Chronic pain (sometimes called intractable pain) is pain that lasts more than 6 months. |
| American Chronic Pain Association^16^ | Chronic pain can be described as ongoing or recurrent pain, lasting beyond the usual course of acute illness or injury healing, more than 3 to 6 months, and which adversely affects the individual’s well-being. Another definition for chronic or persistent pain is pain that continues when it should not. (IASP 2004) |

**References for Supplementary Table 1**

1. Merskey H, Bogduk N. Classification of chronic pain. 2nd ed (Revised). Seattle: IASP Press, 1994. p 4. <https://www.iasp-pain.org/publications/free-ebooks/classification-of-chronic-pain-second-edition-revised/> Accessed 18 Jan 2023.

2. British Pain Society: Useful Definitions and Glossary. <https://www.britishpainsociety.org/people-with-pain/useful-definitions-and-glossary/> Accessed 17 Jan 2023.

3. Faculty of Pain Medicine: Opioids Aware. <https://www.fpm.ac.uk/opioids-aware-understanding-pain-medicines-pain/about-pain> Accessed 16 Jan 2023.

4. National Institute for Health and Clinical Excellence. NICE guideline [NG193]. Chronic pain (primary and secondary) in over 16s: assessment of all chronic pain and management of chronic primary pain. 2021. <https://www.nice.org.uk/guidance/ng193> Accessed 17 Jan 2023.

5. Scottish Intercollegiate Guidelines Network. SIGN publication no.136. Management of chronic pain. 2013. <https://www.sign.ac.uk/assets/sign136.pdf> Accessed 17 Jan 2023.

6. NHS Inform Scotland. Chronic Pain. <https://www.nhsinform.scot/illnesses-and-conditions/brain-nerves-and-spinal-cord/chronic-pain> Accessed 17 Jan 2023.

7. NHS Kernow CCG (NHS Cornwall and Isles of Scilly): RMS Clinical Referral Guidelines: Pain Services (2020). <https://rms.cornwall.nhs.uk/primary_care_clinical_referral_criteria/pain_services> Accessed 17 Jan 2023.

8. Wigan Borough CCG: Opioid Prescribing for Chronic Pain: Resource Pack. 2018. <https://gmmmg.nhs.uk/download/?file_id=184> Accessed 17 Jan 2023.

9. Oxford University Hospitals NHS Foundation Trust: Optimise. [https://www.ouh.nhs.uk/optimise/information/](https://www.ouh.nhs.uk/optimise/information/%20)  Accessed 17 Jan 2023.

10. Institute for Chronic Pain: What is Chronic Pain (2012)? <http://www.instituteforchronicpain.org/understanding-chronic-pain/what-is-chronic-pain> Accessed 17 Jan 2023.

11. Institute of Medicine (US) Committee on Advancing Pain Research, Care, and Education. Relieving Pain in America: A Blueprint for Transforming Prevention, Care, Education, and Research. Washington (DC): National Academies Press (US); 2011. p.33. <https://nap.nationalacademies.org/read/13172/chapter/3#32> Accessed 17 Jan 2023.

12. The Pain Toolkit Persistent Pain: What is Pain? [https://www.paintoolkit.org/persistent-pain/what-is-it](%20https://www.paintoolkit.org/persistent-pain/what-is-it) Accessed 17 Jan 2023.

13. American Society of Anesthesiologists. Made for This Moment: Chronic Pain.<https://www.asahq.org/madeforthismoment/pain-management/types-of-pain/chronic/> Accessed 17 Jan 2023.

14. National Institute of Neurological Disorders and Stroke: Chronic Pain (2022). <https://www.ninds.nih.gov/health-information/disorders/pain> (Accessed 17 Jan 2023.

15. Tennant, F. Types of Chronic Pain (2015).[https://www.practicalpainmanagement.com/patient/resources/understanding-pain/types-chronic-pain .](https://www.practicalpainmanagement.com/patient/resources/understanding-pain/types-chronic-pain%20.) Accessed 17 Jan 2023.

16. American Chronic Pain Association: Chronic Pain. [https://www.theacpa.org/conditions/chronic-pain/](https://www.theacpa.org/conditions/chronic-pain/%20.)  Accessed 16 Jan 2023.
